# *H*-index in medicine is driven by original research

**DOI:** 10.3325/cmj.2018.59.25

**Published:** 2018-02

**Authors:** Jan K. Nowak, Karol Lubarski, Lukasz M. Kowalik, Jaroslaw Walkowiak

**Affiliations:** Department of Pediatric Gastroenterology and Metabolic Diseases, Poznan University of Medical Sciences, Poznan, Poland

## Abstract

**Aim:**

To investigate the contribution of selected types of articles to *h-*indices of medical researchers.

**Methods:**

We used the Web of Science to export the publication records of various members from 26 scientific medical societies (13 European, 13 North American) associated with 13 medical specialties. Those included were presidents (n = 26), heads of randomly chosen committees (n = 52), and randomly selected members of those committees (n = 52). Publications contributing to *h*-index were categorized as research articles, reviews, guidelines, meta-analyses, or other published work.

**Results:**

Overall, 3259 items authored by 129 scholars were analyzed. The median *h*-index was 19.5. The median contribution of research articles to *h*-index was 84.4%. Researchers in the upper *h*-index tercile (≥28.5) had a larger share of research articles that contributed to *h*-index in comparison with those in the lower *h*-index tercile (≤12.5) (median 87.3% [1st-3rd quartile: 80.0%-93.1%] vs 80.0% [50.0%-88.9%], *P* = 0.015). We observed an analogous difference with regard to guidelines (1.1% [0%-3.7%] vs 0% [0%-0%], *P* = 0.007).

**Conclusions:**

Original research drives *h*-indices in medicine. Although guidelines contribute to *h*-indices in medicine, their influence is low. The specific role of randomized controlled trials in building *h*-index in medicine remains to be assessed.

Within a decade since Jorge E. Hirsch proposed *h*-index as a research output measure (1), its use has spread throughout the global scientific community. Today, this measure not only indicates accomplishment, but – by co-defining it – it also influences our behavior. *H-*index, which seems to be easy to interpret, concurrently carries and conceals the complexities of the reality it describes (2).

In medicine, certain types of citable documents contributing to an author’s *h-*index do not represent original research, ie, primary investigation of real-life phenomena to produce new knowledge. These publications include guidelines, consensus statements, and meta-analyses, which all tend to be cited often (3). As Dimitris Tousoulis and Christodoulos Stefanadis (4) noted, this presents an important problem when assessing an author’s research activity.

Therefore, we hypothesized that the *h*-index in medicine is largely influenced by citations of documents that do not report original research but rather reflect clinical accomplishment instead. The aim of this cross-sectional study was to investigate the contribution of selected types of articles including research articles, reviews, guidelines, meta-analyses, and other works, to the *h-*indices of medical researchers.

## MATERIAL AND METHODS

### Material

We selected 13 medical specialties for which both European and North American scientific society existed and had information on committee chairs and other members available on their webpages. The selected specialties included anesthesiology (European Society of Anaesthesiology, American Society of Anesthesiologists), cardiology (European Society of Cardiology, American College of Cardiology), dermatology (European Academy of Dermatology and Venereology, American Academy of Dermatology), endocrinology (European Society of Endocrinology, The Endocrine Society), gastroenterology (European Society for Gastrointestinal Endoscopy, American Gastroenterological Association), hematology (European Hematology Association, American Society of Hematology), neurology (European Academy of Neurology, American Academy of Neurology), ophthalmology (European Society of Ophthalmology, American Academy of Ophthalmology), pediatric gastroenterology (European Society for Paediatric Gastroenterology, Hepatology and Nutrition, North American Society for Pediatric Gastroenterology, Hepatology and Nutrition), psychiatry (European Psychiatric Association, American Psychiatric Association), radiology (European Society of Radiology, American College of Radiology), thoracic surgery (European Society of Thoracic Surgeons, American Association for Thoracic Surgery), and urology (European Association of Urology, American Urological Association).

### Method

In each of the 26 societies, we identified five persons including the president of the society, two chairpersons of scientific or medical committees, and two members thereof. Computer-generated pseudorandom numbers were used to select two committees per society and one member per committee. Thus, 130 researchers were included in the study: 26 presidents, 52 chairmen or chairwomen, and 52 committee members.

In March 2017 for the Endocrine Society, January 2018 for the cardiological societies, and November 2015 for all other societies included in the study, we used the Web of Science’s Author Search (Thomson Reuters, Toronto, Canada; Clarivate Analytics, Philadelphia, USA) to identify manuscripts published by each researcher. The provision of the field of research and the home institution(s) helped narrow the results. After downloading the full publication record, including the number of citations per article, we also obtained the *h*-index and the total number of citations with and without self-citations calculated by the Web of Science. Additionally, we defined “time since first publication” as a time span starting in the year when first articles by the author were published and ending at the time of our search (we excluded outlying single publications).

Our focus was on publications contributing to the *h*-index (5). To classify articles, we established five categories as follows: research articles, reviews, guidelines, meta-analyses, and other published work (encompassing case reports, editorials, journal correspondence, and so on). Each item was categorized by at least two of the authors (JKN, KL, and LMK) who needed to reach agreement on the article type. In most cases, the type of article was evident from the title. Where this was not clear enough, abstracts or full texts were accessed. Classification of the article by the Web of Science was used as a supplementary information source.

### Statistical analysis

The contribution of selected types of articles to *h-*indices of medical researchers has not been investigated in detail, so we determined the sample size by considering our capabilities. The subgroup size of 24 people was sufficient to detect the following difference in the contribution of a category of article to *h-*index: between 20% in one group and 40% in another, given the standard deviation of 25%, the power of the test 76% (double sided, continuity correction), and the alpha level 0.05.

We calculated the percentages of publications from each category among publications contributing to *h*-index of each researcher. We then analyzed the data for the total sample and for scholars grouped by *h-*index terciles. Since Kolmogorov-Smirnov test showed that the distribution of values was not normal, we applied the Mann-Whitney U test to compare the relative contribution of the types of publications to *h-*indices of researchers belonging to the upper vs the lower *h*-index tercile. Data were presented as medians with 1st-3rd quartile range and 95% confidence intervals (CI). All other analyses were exploratory, including the calculation of Spearman’s rank-sum correlations and a forward stepwise regression used to compensate for confounding.

We used STATISTICA 12 and 13 (StatSoft Inc., Tulsa, USA; TIBCO, Palo Alto, USA) to perform statistical tests and considered *P* < 0.05 as statistically significant.

## RESULTS

**Figure 1:**
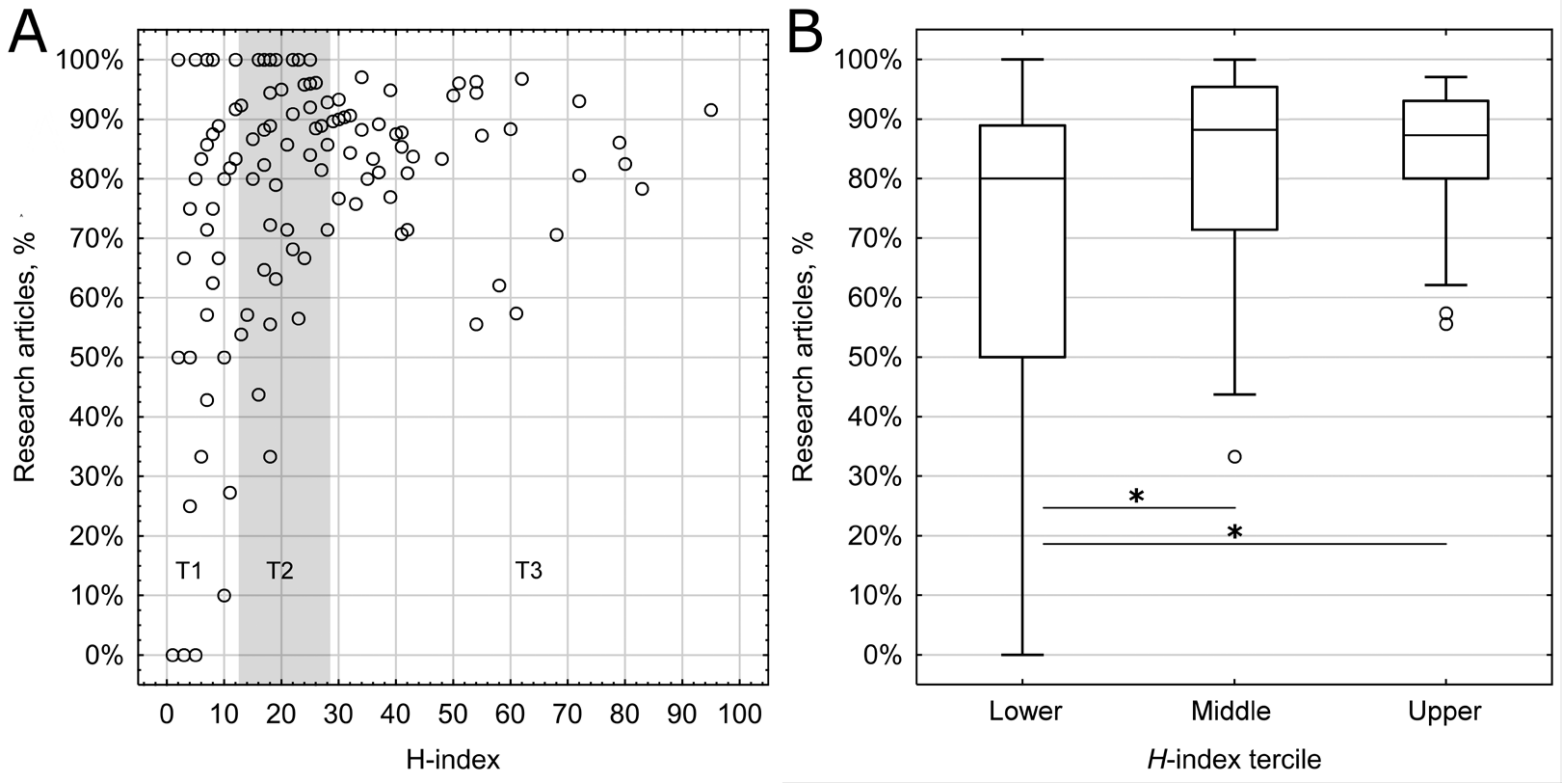
Contribution of research articles to *h*-index. **A.** Scatterplot of percentage of research articles among publications contributing to *h*-index of members of selected medical societies vs their *h*-index with terciles indicated (T1, T2 – shaded area, T3). **B.** Boxplot of the same percentage, by *h*-index tercile. Medians, 1st – 3rd quartile ranges, non-outlier ranges and outliers (circles) are shown; *P* < 0.05 (*).

Overall, 3259 items published by 129 researchers were analyzed; one person did not author any publications (Online Resource 1). Presidents of societies had the highest *h*-index, number of citations, and percentage of self-citations (Table 1). A similar association between *h*-index and self-citations was found when the authors were grouped by *h*-index tercile (Table 2). Median time since first publications was significantly higher in the upper than in the lower *h*-index tercile. Analysis of contributions of different types of articles to the *h-*index showed that research articles accounted for the greatest percentage (Table 3; Figure 1A and 1B). Although the majority of reviews was authored by scholars with at least average *h-*index, some of the scientists with low *h*-indices had a relatively high contribution of reviews; this was evidenced by the high upper quartile (20%) of review papers among items contributing to *h*-index.

**Table 1:** Citation measures and time since first publications according to the current roles of researchers in their scientific societies

		Citation measures (median, 1st-3rd quartile range)			
Population	N	*H*-index	total citations (n)	self-citations (%)	time since first publications (years)
Total	130	19.5 (10.0–34.0)	1545 (413–4405)	4.6 (1.6–7.2)	25 (17–31)
Presidents of societies	26	40.5 (17.0–58.0)	5567 (1125–13690)	5.1 (1.7–7.0)	31 (26–37)
Chairwomen and chairmen of committees	52	18.5 (11.0–32.5)	1560 (504–4239)	4.4 (1.2–7.8)	24 (17–30)
Members of committees	52	17.5 (7.0–28.5)	1208 (239–3536)	4.4 (1.9–7.0)	22 (17–28)

**Table 2:** Characteristics of researchers classified by *h*-index terciles and a comparison of percentage of self-citations and time since first articles published between the researchers categorized into the upper and the lower tercile

		Measures (median, 1st–3rd quartile range)			
Population per *h*-index tercile	N	*H*-index	total citations (n)	self-citations (%)	time since first publications (years)
upper (≥28.5)	43	42.0 (34.0–58.0)	6860 (4405–13690)	6.5 (4.8–11.0%)	31 (24–36)
middle (12.5–28.4)	44	19.5 (17.0–24.5)	1545 (1111–2158)	4.7 (2.2–6.7%)	25 (19–31)
lower (<12.5)	43	7.0 (4.0–10.0)	243 (105–423)	1.2 (0.2–4.0)	17 (11–25)
*P* (upper vs lower tercile)				<0.001	<0.001

**Table 3:** Percentages of publication categories contributing to *h*-indices of members of medical societies. Percentages of article types were compared between the upper and lower terciles of *h*-index

	Publications per category (%; median, 1st-3rd quartile range)				
Population	Original research	Reviews	Guidelines	Meta-analyses	Other
Total	84.4 (71.4–92.3)	6.3 (0–14.3)	0 (0–3.7)	0 (0–0)	0 (0–7.3)
Per *h*-index terciles					
upper (≥28.5)	87.3 (80.0–93.0)	6.7 (3.0–14.0)	1.1 (0–4.0)	0 (0–0)	2.0 (0–5.0)
middle (12.5–28.4)	88.2 (71.4–95.4)	5.9 (0–10.8)	0 (0–5.6)	0 (0–0)	0 (0–5.3)
lower (≤12.5)	80.0 (50.0–88.9)	0 (0–20.0)	0 (0–0)	0 (0–0)	0 (0–20.0)
*P* (upper vs lower tercile)	0.015	0.487	0.007	0.532	0.369

**Figure 2:**
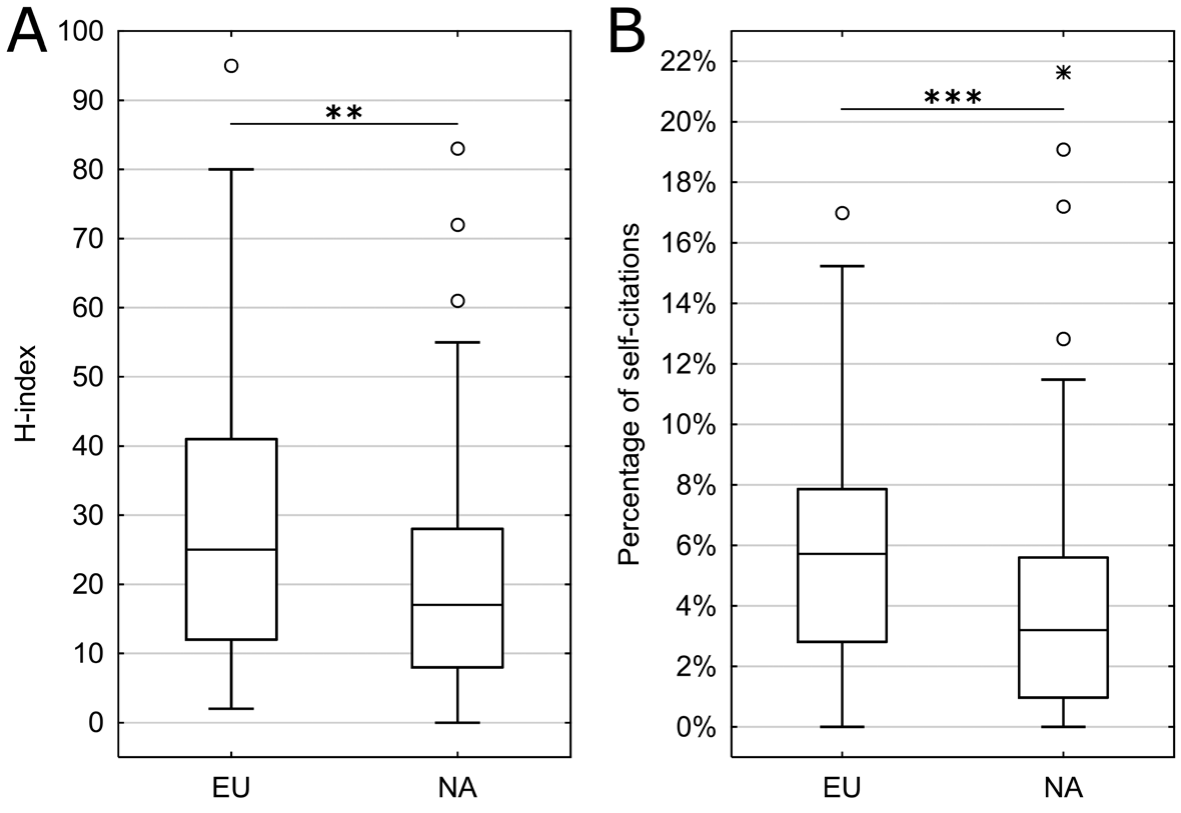
Comparison between European and North American researchers. **A.** *H*-index compared between members of selected European (EU) and North American (NA) medical societies. **B.** Percentage of self-citations compared between members of selected EU and NA medical societies. Medians, 1st – 3rd quartile ranges, non-outlier ranges, outlier (circles) and extreme value (star) are shown; *P* < 0.01 (**), *P* < 0.001 (***).

**Figure 3:**
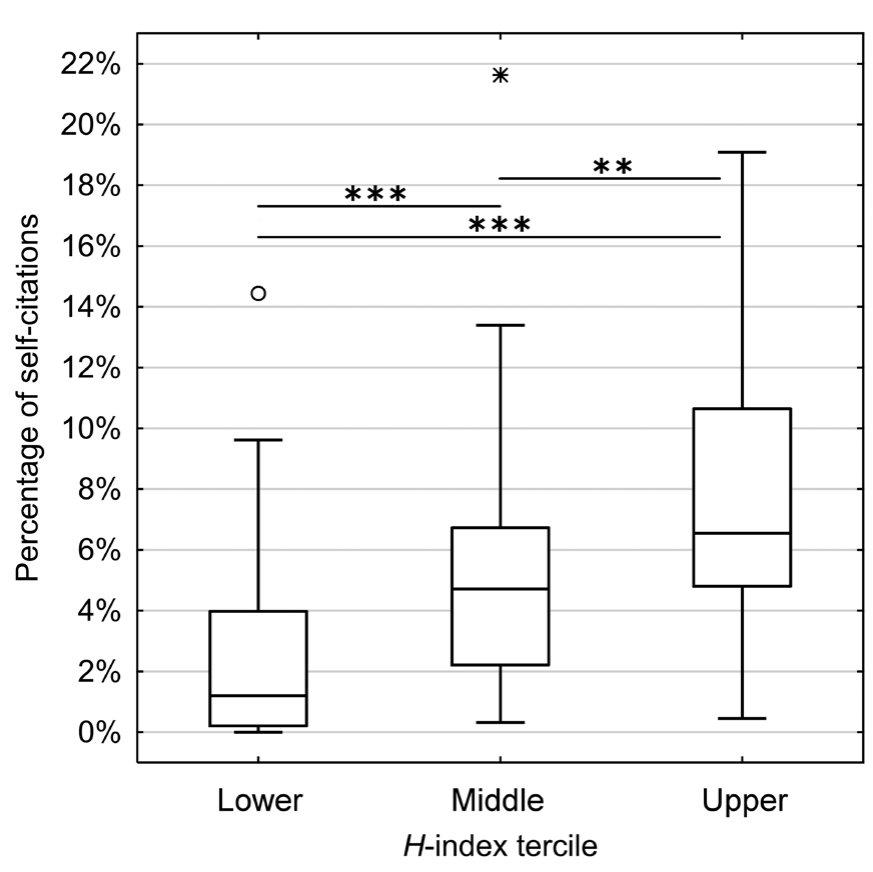
Overall percentage of self-citations by members of selected medical societies grouped by *h*-index tercile. Medians, 1st-3rd quartile ranges, non-outlier ranges, outlier (circle), and extreme value (star) are shown; *P* < 0.01 (**), *P* < 0.001 (***).

An unexpected findings from our exploratory analyses was that European researchers seemed to have higher *h-*indices than their North American peers (25 [12-41] vs 17 [8-28], *P* = 0.006; Figure 2A) and this did not seem confounded by time since first publications (*P* = 0.549), which moderately correlated with *h*-indices (ρ = 0.53, *P* < 0.001). However, the European authors had a greater percentage of self-citations (5.7% [2.8-7.9] vs 3.2% [1.0-5.6], *P* = 0.001; Figure 2B). The correlation between *h*-index and the percentage of self-citations was also moderate (ρ = 0.55, *P* < 0.001; Figure 3). The percentage of research articles was greater in European researchers’ *h*-index publications (88.2% [80.6–94.4%] vs 80.0% [63.9–89.3%]; *P* = 0.009). There were no other differences in article type frequencies in *h*-index between the two regions. A forward stepwise multivariable regression analysis including the continent, self-citation rate, and research article percentage suggested that only the latter two factors were associated with higher *h*-indices (adjusted R^2^ = 0.25, model *P* < 0.001), ie, self-citation rate (β = 0.44 [95% CI 0.29–0.59]) and research article percentage (β = 0.21 [95% CI 0.06–0.36], whereas no independent effect of the continent was found.

We also found that 44.6% (n = 58) of the investigated medical researchers had an *h-*index at least equal to the number of years since first publications. This percentage was as follows in subgroups: 10-19 years 38.2% (n = 34), 20-29 years 50.0% (n = 48), 30-39 years 43.2% (n = 37). When we compared this group of researchers with those who did not meet the above criterion, we found a modestly larger percentage of guidelines (1.1% [0–4.9] vs 0% [0–0], *P* = 0.029) and reviews (8.0% [3.2–15.3] vs 4.4% [0–14.3], *P* = 0.040) among articles contributing to *h*-index.

## DISCUSSION

To the best of our knowledge, this is the first study to measure the influence of different publication categories on *h*-indices in medicine. Our main finding was that, among the members of selected medical societies, *h*-indices were driven by original research.

Our choice of article types reflects the initial observation that stimulated us to perform this study. We observed a general trend to publish guidelines and meta-analyses and that these documents are cited remarkably well because of their clinical usefulness (6). Systematic reviews and meta-analyses have a mean annual citation rate of about 7 per item (3,7). *H-*index is a measure often perceived as reflecting research accomplishment and, as such, may influence how basic research funding is distributed in biomedicine. Our hypothesis was that citations to the aforementioned document types could give some scholars an advantage. Our impression was incorrect and calculating the *h-*index for original research only for these purposes would not be useful.

We also found that guidelines were authored by more accomplished researchers who already have considerable *h*-indices. This probably adds to the nonlinearity of this metric by slightly accelerating the increase in *h*-index after certain recognition and position are achieved. For some authors within the lower *h*-index tercile, contribution of review papers and other publications, such as case studies, was significant. One-third of the researchers in the lower *h*-index group had no more than 50% of their *h*-index built by citations to original research.

The median *h*-index in the sample was 19, suggesting a high academic rank of the selected scholars (8-11). Clearly, this was not representative of members of societies who were not involved in committees, who probably had lower citation indices. For instance, American and British specialty surgeons rarely have *h*-indices exceeding 10 (12,13). Furthermore, the median time since first publication was 26 years. Thus, the generalizability of our study results is limited to more experienced members of scientific societies in disciplines in which clinical practice is usually associated with research.

The positive association between *h*-index and self-citation percentage cannot be explained by a putative existence of a manipulation through strategic self-citation (14). Self-citations are unlikely to increase an author’s *h*-index (15,16). It was also suggested that original research publications tend to include more self-citations, and that there is no difference in self-citation rates between the United States and other countries (17). Therefore, self-citations are necessary to refer to previously described methods and findings, upon which the new studies are built (18).

Another finding from exploratory analyses is that an *h*-index at least equal to the number of years since first publications (*m*-index = 1) may be a good rule of thumb to indicate excellent citation achievement in medicine (19). This would not apply to all specialties equally. For example, only 10% of academic emergency physicians obtain an annual *h*-index increase of 0.5 (20). While a high *h*-index indicates accomplishment, the opposite is not always true (1).

The similarity of *h*-indices of committee heads and members seems counterintuitive. However, the two groups did not differ in time since first publications. It seems that a high rotation of scholars occupying the posts of committee heads along with a general high level of achievement in all the committee members could explain the above finding.

The finding that Europeans had higher *h*-indices and/or self-citation rates is puzzling. We did not expect to find differences in this respect. It may be that the activity of members of European medical societies’ committees is more research-oriented than it is the case in North America. This might warrant further study.

Our choice of the Web of Science was determined by data export capabilities of this tool. We would have preferred Scopus (Elsevier, Amsterdam, Netherlands) because of its efficient author identification algorithm (21). However, we encountered problems applying our methods to files obtained from Scopus. We did not consider Google Scholar (Google Inc., Mountain View, United States), since it inflates the *h*-index (22) by the factor of 1.4 compared to the Web of Science (23).

With respect to the limitations of our study, our selection of medical specialties was biased toward the internal medicine. Although wanted to choose a wider range of specialties, in many cases we were unable to find the necessary information to implement our protocol. We chose presidents, heads of committees, and members of committees at a 1:2:2 ratio to obtain a sample of researchers with a wide spectrum of *h-*index values. This is also the reason for choosing both European and North American societies – it was not our intention to compare societies from the two continents, but to obtain a reasonable sample. The sample size, although moderate, proved to be sufficient to demonstrate significant trends.

The identification of publications, which were authored by chosen scholars, carried a risk of error. Indeed, it is currently not possible to obtain an exact list of a researcher’s works without their aid. Therefore, we strived to assure the integrity of the data at two levels: in the Web of Science and while classifying articles. Where publications were not consistent with a scientist’s profile, they were manually verified.

We used an extreme groups approach in the main analysis. Comparing the upper and the lower terciles was justified by data nonlinearity and the limitation imposed on sample size by the long time required to include a researcher in the study (Preacher et al. 2005). By increasing power this method allows for cost-effective identification of potential effects and their general directions. Taking into account the limitations of such a design and the nature of the results—including the higher upper quartile for the percentage of guidelines in the middle than in the upper *h*-index tercile—we draw our conclusions principally from the descriptive statistics of our data.

It is true that categorizing a random sample of publications not contributing to *h*-index could reveal interesting patterns. This was, however, beyond the scope of our study, just as were comparisons between the specialties with insufficient sub-sample size. Proportional sampling of the committees, which might have reduced the selection bias, was beyond our capabilities.

The *h*-index is today what its inventor wanted it to be: “a useful yardstick” that often proves useful (24). The main disadvantages of the *h-*index were already listed by Jorge E. Hirsch in 2005 (1). They were further evaluated since and include a lack of influence of an author’s position on an article on the metric (25,26), ignorance of the skewness of the citation distribution, and field- and age-dependence—most of which are inherent to citation-based benchmarks (27-29). Various alternative metrics were proposed (30), including the Pagerank-Index (31). Aggregate use of metrics may lead to a more nuanced discrimination of researchers (32). However, the employment of any indicators will always need to be supplemented by the awareness of both their strengths and their shortcomings (30,33,34).

Original research drives *h*-indices in medicine. Although guidelines contribute to *h*-indices in medicine, their influence is low. The role of randomized controlled trials in building *h*-index in medicine remains to be assessed.
